# Supplementary material for: Inflammatory Myofibroblastic Tumor of the Upper Airways Harboring a New TRAF3-ALK Fusion Transcript
Source: Children (Basel). 2021 Jun 15;8(6):505. doi: 10.3390/children8060505 (PMC8232338; doi:10.3390/children8060505)
Supplement: Supplementary file 1 [file children-08-00505-s001.zip › children-1248893-supplementary.pdf]

Supplementary Materials

Table S1. Cases of Children with IMT of the Upper Airway in the Literature.

References (year)	*Patient (gender and age)*	*Localization*	*Hystological assessment*	*Immuno Isto chemistry*	*Treatment*	*Follow-up (time)*
Zapatero et al(1989)[11]	M, 6 y	Endotracheal	Inflammatory pleomorphic cellular proliferation with intermixed lymphocytes, plasma cells, fibroblasts, and capillary neoformation	NR	Surgical resection	CR (8 y)
Aijaz et al(1994)[12]	M, 8 y	Endotracheal	Spindle cells with plasma cells, histiocytes, foamy macrophages, neutrophils, and some eosinophils	Vimentin +, desmine focal +, ALK NR	Surgical resection	CR (NR)
Storck et al (1995)[13]	F, 16 y	Right main bronchus	Spindle cells with an inflammatory cell infiltrate	NR	Sleeve resection	CR (8 y)
Denwar et a(1997)l[14]	M, 10 m	Endotracheal	Proliferation of spindle cells (myoblasts and fibroblasts) and inflammatory cell infiltrate	NR	Surgical resection	NR
Bumber et al(2001)[15]	M, 14 y	Endotracheal	Areas of spindle cells sparkled with inflammatory cells	Vimentin ++, SMA +/-, ALK NR	Surgical resection and CO_2 laser	CR (1 y)

Chan et al(2003)[16]	F, 7 y	Right main bronchus	Fibrous tissue with areas of necrosis, lymphocytic infiltration, and plasma cells	NR	Biopsy and COX2-Inhibitor	CR (8 m)
Hoseok et al(2005)[17]	M, 4 y	Carina and left main bronchus	Proliferation of spindle-shaped fibroblasts and myofibroblasts arrayed in fascicles with some storiform architecture	SMA +, vimentin +, ALK NR	Surgical resection with carinal reconstruction	CR (3 m)
Sinvadan et al(2007)[18]	F, 9 y	Endotracheal	Sprinkling lymphocytes and spindle cells	SMA, vimentine +; ALK NR	Surgical resection	CR (NR); tracheostomy for subglottic stenosis
Venizelos et al(2008)[19]	M, 13 y	Endotracheal	Spindle-shaped cells with ovoid nuclei, sparse chromatin, and eosinophilic cytoplasm	Vimentine and ALK +	Surgical resection	CR (6 m)
Breen et al(2008)[20]	F, 11 y	Left main bronchus	Fusiform components, inflammatory cells, and bronchial gland	NR	Surgical resection	CR (3 y)
Lizarbe et al(2009)[21]	M, 8 y	Paratracheal	Myxoid areas with fibroblast cells, epithelioid cells, and histiocytes mixed with inflammatory cells	Vimentine +,desmina, SMA, ALK -	Surgical resection	PD

NR: Not Reported. CR: Complete Remission

Ochs et al(2010)[22]	F, 5 y	Left main bronchus	Spindle cells with a palisading pattern (arrow) and admixed leukocytes	NR	Endobronchial resection	CR (3 y)
Sacco et al (2010)[23]	F, 6 y	Paratracheal	Spindle cells surrounded by a collagenous stroma	Vimentin +, SMA + (focal), EMA + Clusterin +/-<br><br>ALK -	Subtotal resection + low-dose CT (vinblastine + MTX)	PR (16 months)
Brodie et al (2011)[24]	F, 11 y	Endotracheal	Spindle cells with inflammatory background	ALK -	Surgery of the bulk mass and laser	CR (2 y)
	F, 15 y	Left main bronchus	EMA +, dense spindle cell proliferation	ALK +	Sleeve resection of the left mainstem bronchus	CR (3 y)
El-Desoky et al(2013)[25]	F, 9 y	Left main bronchus	Spindle cells with mild atypia	EMA+, Vimentina +, SMA+ ALK NR	Left lobe Lobectomy	CR (NR)
